# A review of robotic-assisted laparoscopic partial nephrectomy in the management of renal duplication anomalies

**DOI:** 10.3389/fsurg.2024.1364246

**Published:** 2024-02-09

**Authors:** Nikhil Varun Batra, Pankaj Dangle

**Affiliations:** Riley Hospital for Children at IU Health, Indianapolis, IN, United States

**Keywords:** robotic surgery, renal duplication, heminephrectomy, partial nephrectomy, minimally invasive surgeries (MIS)

## Abstract

Open and purely laparoscopic partial nephrectomy or heminephrectomy has been the standard management for renal duplication anomalies for symptomatic children with non-functional renal moieties. While robotic-assisted laparoscopic partial nephrectomy (RALPN) has been established as a safe and feasible option for the management of renal duplex anomalies, there remains a paucity of data on this topic. The aim of this study is to comprehensively review all available outcomes data and update the use of emerging technologies in robotic surgery which continue to make RALPN a viable and advantageous option in the management of renal duplex anomalies.

## 1 Introduction

While upper and lower tract reconstructions are reasonable options for the management of renal duplication anomalies, open and purely laparoscopic extirpative upper tract procedures have long been performed on children who are symptomatic with non-functional moieties. Laparoscopy in general has several advantages over open surgery including lower blood loss, short length of stay, and decreased postoperative narcotic use, but is known to be operatively challenging in the case of pediatric partial nephrectomy (1, 2). Robotic-assisted laparoscopic surgery has a several advantages to conventional laparoscopy which has made complex reconstruction and extirpative procedures more feasible, including excellent visual magnification with three-dimensional view, improved dexterity and range of motion, among others (3). While robotic-assisted laparoscopic partial nephrectomy (RALPN) has been established as the standard of care of small renal masses in adults, it is much less well studied in pediatric patients in the setting of renal duplication anomalies.

## 2 Discussion

Robotic-assisted laparoscopic partial nephrectomy (RALPN) in children was first described by Lee et al. in 2009 (1). This retrospective study, which evaluated 9 patients who underwent RALPN for a variety of renal duplication anomalies, demonstrated 100% success in their series but with 2 (22%) complications noted (perioperative urinoma requiring percutaneous drain placement and surgical site infection requiring antibiotics) (1). This study reported a mean operative time of 275 min (170–417 min) which was noted to improve over time which the authors attribute to the learning curve for RALPN. This study has since served as the basis for feasibility and safety for RALPN in children, which has been further studied in several larger cohorts and comparative studies.

Subsequent studies have published operative times on RALPN for duplex renal anomalies ranging between 135 and 301 min (4–8). Despite the initial hypothesis by Lee, et al. that operative times may be seen to improve with time and increased experience, it seems likely that some rate limiting elements of the robotic approach (e.g., docking and undocking, troubleshooting ergonomics, etc.) may generally hinder the efficiency of RALPN. A full summary of operative times and outcomes among all included studies in this review can be seen in Table 1.

**Table 1:** Collective summary of outcomes in RALPN.

Study	Number of patients	Median age years (IQR)	Mean operative time, min (range)	Mean EBL, cc (range)	Success (%)	Mean LOS, days (range)	Reported postoperative complications	Mean follow-up, months (range)
Lee et al. (1)	9	7.2 (0.5–16.5) (mean)	275 (170–417)	49 (5–150)	100%	2.9 (1.9–4.8)	2 (IIIB, II—perc drain, SSI)	6 (0.2–25)
Mason et al. (4)	21	4.1 (0.3–16.7) (mean)	301 (165–526)	36 (min-300)	95% (reoperative rate 4.7%)	1.6 (0.8–3.9)	1 (IIIB—port site hernia)	24 (3–80)
Malik et al. (5)	16	3.1 (0.3–15.6) (mean)	135 (78–201)	10 (5–20)	87% (reoperative rate 13%)	1.9 (1.2–2.8)	2 (IIIA—urinoma)	22.1 (3–56)
Herz et al. (9)	20	3.1 (0.3–10.5)	209 (169–330)	NR	95% (repoerative rate 5%)	NR	1 (IIIB—laparoscopic nephrectomy)	NR
Ballouhey et al. (7)	15	1.7 (0.6–3.3)	201 (130–245)	12 (5–15)	100%	3.4 (1–7)	2 (IIIB, I—port site hernia, asymptomatc fluid collection	46.4 (25–68)
Neheman et al. (8)	18	3.7 (1.4–11.0)	256 (163–458) (median)	5 (0–100)	NR	2 (1–4) (median)	NR	NR

Although reported mean estimated blood loss (EBL) in the initial study by Lee was somewhat high (median EBL 49 cc), more contemporary series have reported much more modest outputs ranging from 5 to 36 cc (4–8). Two comparative studies of RALPN vs. open partial nephrectomy have been published, both of which reported on EBL. Ballouhey, et al. noted no difference in mean EBL between open and robotic cohorts (23 vs. 12 cc) (7). Neheman, et al, however, did note a significant difference between groups, reporting significantly higher median blood loss in open partial nephrectomy vs. RALPN (10 vs. 5 cc, *p* = 0.005) (8). The summary of evidence would at least suggest that RALPN is equivalent, if not better, than an open approach with regards to minimizing blood loss, which is in line with classically known benefits to minimally invasive surgery (MIS).

Although several studies report on a number of postoperative outcomes measures, the two aforementioned comparative studies are more useful in weighing surgical approaches in the management of duplex renal anomalies and are worthy of mention in more detail. Ballouhey, et al. evaluated 15 children who underwent RALPN and 13 who underwent open partial nephrectomy. They found no difference in operative time (201 vs. 178 min), complications (2 vs. 2), and pre- and postoperative renal differential function (−3% vs. −5%) in RALPN vs. open cohorts. Postoperative LOS was significantly shorter (3.4 vs. 6.3 days, *p* < 0.001) in RALPN vs. open groups (7). One patient in the RALPN cohort required a secondary procedure to address a port site hernia. No other secondary procedures or open conversions were required. No secondary procedures were required in the open group. Neheman, et al. offered a comparison between open partial nephrectomy, laparoscopic partial nephrectomy, laparoendoscopic single site (LESS) partial nephrectomy, and RALPN. They included 24 patients that underwent an open approach, 7 laparoscopic, 10 LESS, and 18 robotic partial nephrectomies for duplex renal anomalies. Preoperative characteristics were similar between groups. Open partial nephrectomy was found to have significantly lower median operative time than RALPN (154.5 vs. 256 min, *p* = 0.005) (8). Interestingly, RALPN was significantly longer than a LESS approach (256 min vs. 140 min, *p* = 0.005) but not a purely laparoscopic approach. An open approach was seen to have significantly higher median blood loss than all other MIS groups, including RALPN (10 vs. 5 cc, *p* = 0.005). Median LOS was also significantly longer for open approach vs. all other groups, again including RALPN (3 vs. 2 days, *p* = 0.005) (8). There were 6 complications amongst all groups, all of which were Clavien-Dindo grade 2. Six patients amongst all groups underwent secondary procedures (four Deflux and two ureteral stump excisions), although the original surgical approach at the time of partial nephrectomy is not specified in this study.

Postoperative analgesic use has been studied in several series. Morphine equivalent intake following RALPN has been reported to be 0.18–0.4 mg/kg/day (1, 5). Both series comparing RALPN to open partial nephrectomy have noted significant reductions in postoperative analgesic use. Ballouhey, et al. reported a morphine equivalent intake of 1.08 mg/kg/day vs. 0.52 mg/kg/day following open vs. RALPN, respectively (*p* < 0.001) (7). Neheman, et al. reported a reduction in both groups overall but a similar disparity of postoperative morphine equivalent intake of 0.554 mg/kg/day vs. 0.039 mg/kg/day in open and RALPN groups, respectively (*p* = 0.005) (8). The summary of evidence regarding postoperative analgesic use seems to favor RALPN over an open approach.

Regarding reduction in renal function following RALPN, few studies report on this parameter as postoperative nuclear renography is not standardly obtained, barring a postoperative issue. Malik, et al. reported that seven out of their 16 patients that underwent RALPN had pre- and postoperative nuclear renography obtained demonstrating a mean change in renal function of −4.7% (5). This was very similar to that reported by Ballouhey, et al. at −4.7% following RALPN (7).

Operative success, defined as ability to complete surgery robotically or absence of reoperation for primary or secondary diagnosis or postoperative complication, was reported in 6 of the 7 studies included in this review for a range of 87%–100% and is summarized in Table 1 (1, 4–7). High-grade postoperative complications (Clavien Dindo grade 3 or greater) are infrequent following RALPN and are also summarized in Table 1 for the series included in this review (1, 4–8). Systematic review by Aksenov, et al. has evaluated complications in pediatric urologic MIS, including RALPN. They included 5 studies with 99 patients meeting their criteria and noted a rate of 6.06% Clavien IIIa or IIIb complications in RALPN (10). Compared to other minimally invasive modalities (pure laparoscopic or laparoendoscopic single site surgery), they found no statistical difference in the rate of complications. No patients who underwent RALPN required open conversion and there were no Clavien IV or V complications. The authors reported that only 35.8% of all included studies in their review used Clavien-Dindo classification to report complications which they cite as a limiting factor in determining the true rates of complications in pediatric urologic MIS cases (10).

Often reported in the literature as a potential problem that requires either intervention or closer surveillance are the development of postoperative fluid collections or urinomas. Lee, et al. reported one of nine patients (11%) developed an asymptomatic urinoma (1). The patient in their series was treated with percutaneous drainage at the request of the parents. The subsequent series report a range of 0%–29% of patients who developed postoperative fluid collections or urinomas, none which required percutaneous drainage of any type (4–8). Although interesting that in the RALPN literature that all reported urinomas were asymptomatic and only one required intervention, it is known from the open and laparoscopic partial nephrectomy literature that these can result from unrecognized collecting system injuries or residual functional parenchyma and intervention may be warranted in symptomatic cases or in those with extenuating circumstances (1, 9).

Injury to the unaffected renal moiety during heminephrectomy is estimated to be around 4%–5% regardless of open or laparoscopic surgical approach (11). Of all series evaluated in this review, only one patient required completion nephrectomy for an unrecognized vascular injury to the ipsilateral unaffected renal moiety at the time of primary surgery (6). Reportedly, this patient had no follow-up issues in subsequent surveillance (6). Preoperative imaging can be helpful in elucidating duplex renal anatomy and vascular architecture but does not guarantee identification of the complete vascular supply to both renal moieties which may jeopardize the healthy unit. Emerging robotic technologies, like the use of selective arterial mapping (SAM) using indocyanine green (ICG)-aided near-infrared fluorescence (NIRF) imaging, may be beneficial to guide safer dissection and identification of complex anatomy. Herz, et al. evaluated the use of intraoperative SAM in RALPN. They included six patients in their series with a median age of 4.75 years, all of which had duplex renal anomalies (11). Median operative time was 214.5 min, which was comparable to other contemporary series (1, 4–7). They did report an increase in operative time by 16 min with the use of SAM compared to unmatched patients that previously underwent traditional RALPN at their institution (11). They noted in three of six cases (50%) that SAM guided an alternative dissection which would have otherwise put the unaffected renal moiety in jeopardy (11). There were no postoperative complications or secondary surgeries required in this series. The authors report that a single-use vial of ICG only increased the operative cost by $365 in each case provided that the da Vinci robotic surgical system® equipped with a NIRF imaging system is used (11). Although a small study, the use of SAM with NIRF imaging may be a useful adjunct that improves the safety profile of RALPN when the technology is available.

Compared to open partial nephrectomy from an economic standpoint, RALPN is associated with a higher cost despite a shorter length of stay (12). Mahida, et al. utilized pooled national administrative data from the Pediatric Health Information System (PHIS) which includes data from 47 of the largest children's hospitals in the United States to evaluate pediatric patients who underwent robotic surgery during a 5-year period using procedure codes. A total of 56 patients underwent RALPN during the study period. The PHIS system was then used to query patients who underwent corresponding open procedures at the same institution during the study period to serve as a comparison group. Median postoperative length of stay was noted to be 2 days (IQR 1–3 days) in the RALPN group vs. 3 days (IQR 2–4 days) in the open group (*p* < 0.001) (12). Median hospitalization cost was $17,524 in the RALPN group vs. $12,516 in the open partial nephrectomy group (*p* = 0.004) (12).

While minimally invasive approaches to extirpative management of renal duplex anomalies appear to be safe, effective and potentially even advantageous, similar results have been published for robotic reconstructive approaches. In a comparative study, Lee, et al. evaluated open vs. robotic-assisted laparoscopic ureteroureterostomy (RALUU) in duplex renal anomalies. They found similar operative times, EBL, and rates of complications between open and robotic groups with a significantly shorter LOS in the robotic group (1.6 vs. 2.1 days, *p* = 0.04) (13). Ellison and Lendvay reviewed outcomes for 47 patients in 5 studies with duplex renal anomalies who underwent RALUU with a follow-up range between 3 and 16.4 months. Successful outcomes were observed in 95.7% of cases, with two patients in total across all series requiring open revision following RALUU (14). Herz, et al. also reviewed outcomes in RALUU and robotic-assisted laparoscopic common-sheath ureteroneocystostomy (RAL csUN) in their series. They found success rates of 100% across multiple series for RALUU and 72%–100% for RAL csUN (6). Importantly, the follow-up for many of these series is considerably shorter than that seen for RALPN, which may limit the reliability of the reported outcomes given the absence of long-term follow-up. However, despite these limited results, it appears that robotic extirpative and reconstructive approaches to renal duplex anomalies may both be useful tools in the armamentarium for pediatric robotic surgeons that offer many of the traditional benefits of minimally invasive surgery.

In summary, the body of literature on RALPN remains small but supports the notion that this approach is feasible, safe, and has comparable outcomes to open partial nephrectomy for renal duplication anomalies. While operative times and economic costs still favor open partial nephrectomy, comparative studies support a shorter length of stay and reduction in postoperative analgesia requirement in RALPN with one study favoring RALPN for lower EBL as well. While little data exists, the robotic platform harbors the ability to integrate emerging technologies like NIRF imaging which may serve as a useful unique adjunct to RALPN in future study. Future prospective studies with more robust patient numbers would certainly be beneficial in the continued support of RALPN as a viable option in the management of renal duplex anomalies.
